# Supplementary figures and images for: Association of maternal genetics with the gut microbiome and eucalypt diet selection in captive koalas
Source: PeerJ. 2024 May 27;12:e17385. doi: 10.7717/peerj.17385 (PMC11138522; doi:10.7717/peerj.17385)

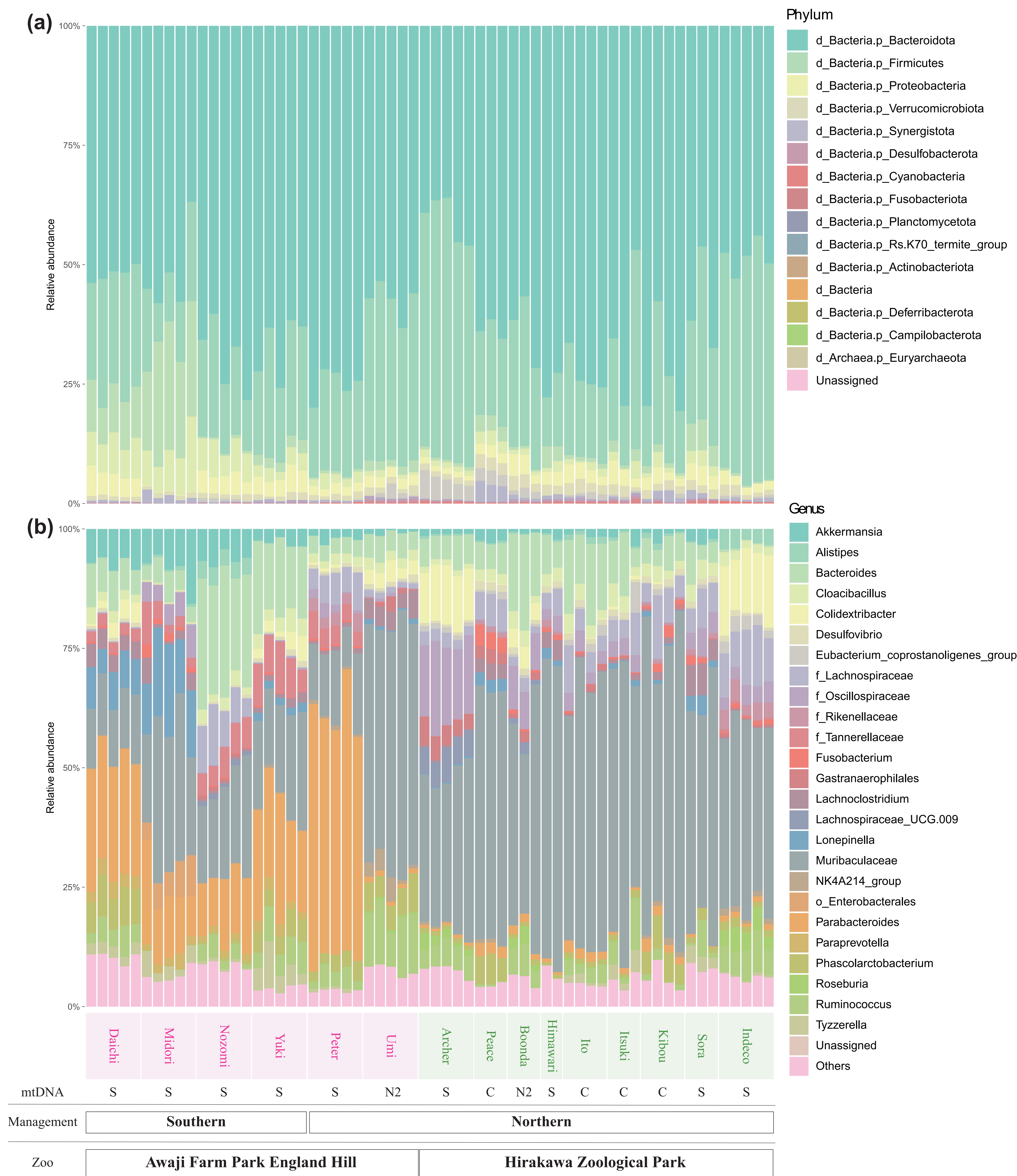

Supplement: Supplemental Information 2 — (a) Relative abundance of gut bacteria at the phylum level. (b) Relative abundance of gut bacteria at the genus level. “Others” include genus that were less than 0.5% of the total abundance and less than 5% in all samples. The mtDNA shows the mitochondrial lineage of each koala (N2, northern 2; C, central; S, southern). The Management shows the management group of each koala. [file peerj-12-17385-s002.pdf]
