# Supplementary material for: Association of maternal genetics with the gut microbiome and eucalypt diet selection in captive koalas
Source: PeerJ. 2024 May 27;12:e17385. doi: 10.7717/peerj.17385 (PMC11138522; doi:10.7717/peerj.17385)
Supplement: Supplemental Information 3 — Principal components analysis of the unweighted UniFrac distances that include individual information (koala names and belonging zoos). [file peerj-12-17385-s003.pdf]

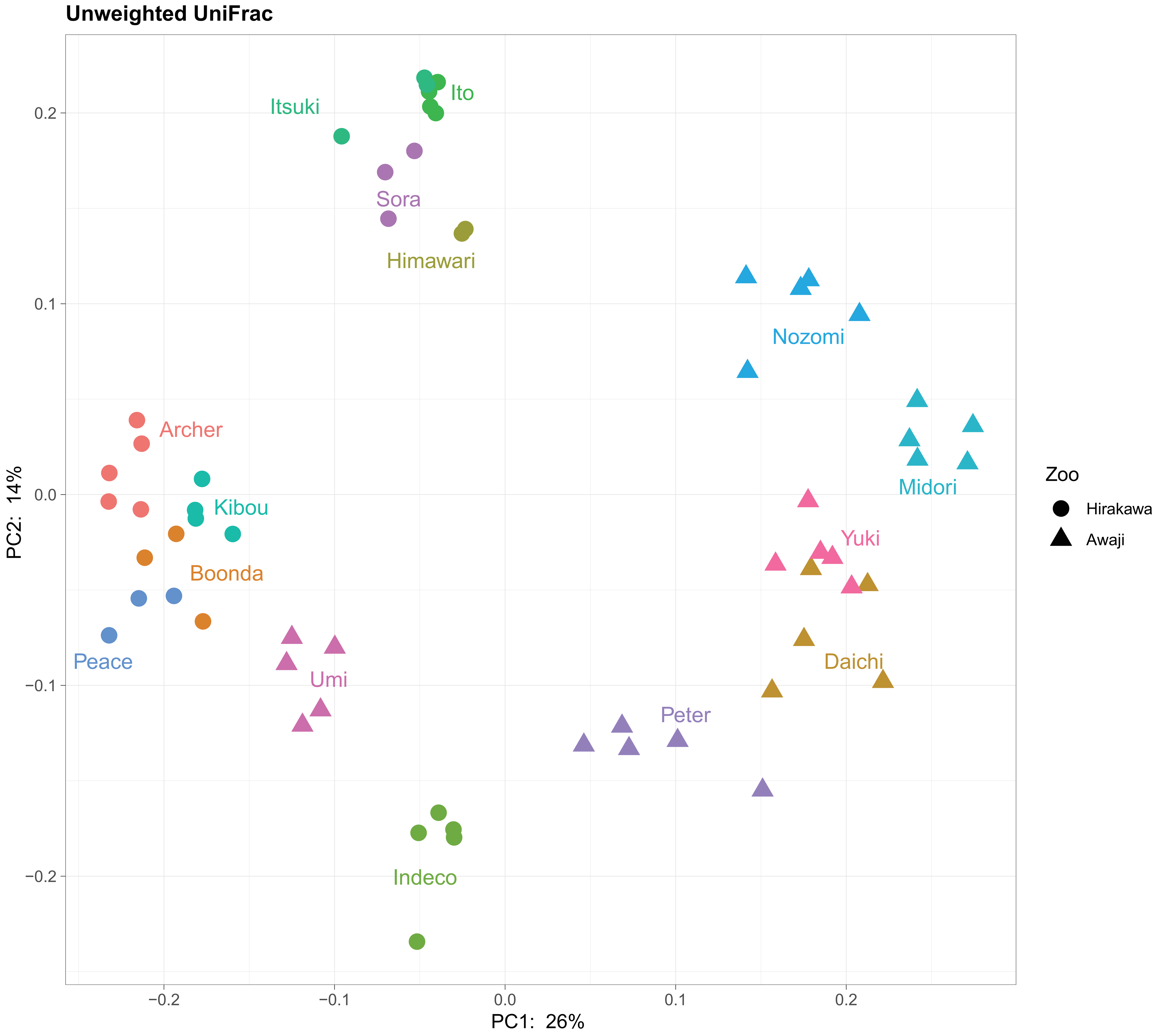

Fig. S2 Principal components analysis of the unweighted UniFrac distances that include individual information (koala names and belonging zoos).
